# Case Report: The Carotid Body in COVID-19: Histopathological and Virological Analyses of an Autopsy Case Series

**DOI:** 10.3389/fimmu.2021.736529

**Published:** 2021-10-26

**Authors:** Andrea Porzionato, Aron Emmi, Martina Contran, Elena Stocco, Silvia Riccetti, Alessandro Sinigaglia, Veronica Macchi, Luisa Barzon, Raffaele De Caro

**Affiliations:** ^1^Department of Neuroscience, Section of Human Anatomy, University of Padova, Padova, Italy; ^2^Department of Molecular Medicine, University of Padova, Padova, Italy

**Keywords:** carotid body, COVID-19, nervous system, silent hypoxia, peripheral arterial chemoreceptors, chemosensitivity

## Abstract

Various authors have hypothesized carotid body (CB) involvement in Coronavirus Disease 2019 (COVID-19), through direct invasion or indirect effects by systemic stimuli (‘cytokine storm’, angiotensin-converting enzyme [ACE]1/ACE2 imbalance). However, empirical evidence is limited or partial. Here, we present an integrated histopathological and virological analysis of CBs sampled at autopsy from four subjects (2 males and 2 females; age: >70 years old) who died of COVID-19. Histopathological, immunohistochemical and molecular investigation techniques were employed to characterize Severe Acute Respiratory Syndrome – Coronavirus 2 (SARS-CoV2) viral invasion and inflammatory reaction. SARS-CoV2 RNA was detected in the CBs of three cases through Real-Time Reverse Transcription Polymerase Chain Reaction (RT-PCR). In these cases, positive immunostaining for Nucleocapsid and Spike protein were also demonstrated, mainly at the level of large roundish cells consistent with type I cells, confirming direct CB invasion. In these cases, T lymphocytes showed focal aggregations in the CBs, suggestive of local inflammatory reaction. Blood congestion and microthrombosis were also found in one of the positive cases. Intriguingly, microthrombosis, blood congestion and microhaemorrages were also bilaterally detected in the CBs of the negative case, supporting the possibility of COVID-19 effects on the CB even in the absence of its direct invasion. SARS-CoV-2 direct invasion of the CB is confirmed through both immunohistochemistry and RT-PCR, with likely involvement of different cell types. We also reported histopathological findings which could be ascribed to local and/or systemic actions of SARS-CoV-2 and which could potentially affect chemoreception.

## Introduction

The possibility of Severe Acute Respiratory Syndrome – Coronavirus 2 (SARS-CoV2) invasion of the carotid body (CB), with consequent chemosensitive impairment, has been hypothesized by various authors in the past months (1–5), as the CB expresses ACE2 (4, 6). The CB and carotid sinus nerve have also been considered as a potential alternative entry way for the solitary tract nucleus. Invasion of CB (3–5) and/or solitary tract nucleus (6) have also been proposed to explain the development of ‘silent hypoxia’ (or ‘happy hypoxia’) in some COVID-19 patients, i.e., severe hypoxemia without signs of respiratory distress (dyspnea) or breathing acceleration (7). Conversely, increased peripheral arterial chemosensitivity and reflex sympatho-activation have also been hypothesized to play a role in COVID-19 patients with comorbidities (8).

Apart from these perspective articles, few empirical data are available about CB in COVID-19 patients. Lambermont et al. detected SARS-CoV-2 in the CB of a 42-year-old COVID-19 patient, through Real-Time Reverse Transcription Polymerase Chain Reaction (RT-PCR) performed on paraffin-embedded sections after glomus tissue microdissection (9). However, histopathological and SARS-CoV-2 immunohistochemical findings were not reported. Conversely, Kantonen et al. (10) analysed CBs from three subjects died of COVID-19, reporting negativity for SARS-CoV-2 Spike-protein immunohistochemistry and absence of pathological findings (6). RT-PCR was not performed (10).

In the present study, we performed an integrated histopathological and virological analysis of CBs of four patients who died for COVID-19.

## Cases Description

The CBs were obtained at autopsy from 4 subjects (2 males and 2 females) who died of COVID-19. All patients were tested positive for Sars-CoV-2 infection by means of RT-PCR performed on naso/oropharyngeal swabs along the clinical course. Clinical and pathological data are summarized in the timeline table (**Supplementary Table 1**). Except for one patient who was comorbidities-free, all others presented pre-existing chronic medical conditions including hypertension (N=3), diabetes (N=1), vascular dementia (N=2), Parkinson’s disease (N=1). One patient died the same day of hospitalization; the other patients were hospitalized for a range of 4-35 days before death occurrence. SARS-CoV-2 infection was furtherly confirmed in all cases by positive post-mortem naso/oropharyngeal swabs and RT-PCR on paraffin-embedded sections from lung samples (threshold cycle values: Case 1, 21.3; Case 2, 35.5; Case 3, 35.3; Case 4, 32.6).

Bilateral CBs were sampled, except for Case 2 (only right CB sampled). In each case, multiple samples from the major organs were also performed for histological examination. CBs sampled from five subjects older than 70 years (age range 71-80; 3 males and 2 females) were also analysed as controls; death occurred at least one year before the COVID-19 pandemic.

Histological and immunohistochemical analyses were performed according to previously published protocols (11, 12). Specimens were fixed in 10% neutral buffered formalin and paraffin-embedded. For each case, histological examination was carried out on five longitudinal sections, 5 µm thick, at 50 µm distance from each other and stained with haematoxylin-eosin and Azan-Mallory.

Immunoperoxidase staining was performed on a Dako EnVision Autostainer according to manufacturer recommendations. Antibodies for CD3 (Polyclonal Rabbit Anti-Human, Dako Omnis, Code Number: GA503), CD20 (Monoclonal Mouse Anti-Human, Clone KP1, Dako Omnis, Code Number: M0814) and CD68 (Monoclonal Mouse Anti-Human, Clone L26, Dako Omnis, Code Number: M0756) were employed to characterize lympho-monocytic infiltrations. Anti-CD61 immunohistochemistry (Monoclonal Mouse Anti-Human, Clone Y2/51, Dako Omnis, Code Number: M0753) was also employed to evaluate the presence of platelet microthrombi. Anti-SARS-CoV-2 nucleocapsid (Rabbit Anti-Human, Sino Biologicals, 40143-R001) and -Spike Subunit 1 Antibody (Monoclonal Rabbit Anti-Human, Clone 007, Sino Biological, Code Number: 40150-R007) immunostainings were employed to evaluate viral tropism within the tissue. Nucleocapsid and Spike antibodies were validated through SARS-CoV-2 infected Vero E6 cells and autopsy-derived lung tissue from SARS-CoV-2 positive patients as positive controls; non-infected cells and lung and CB sections deriving from autopsy cases predating COVID-19 pandemic (2017) were used as negative controls. Peroxidase reactions were repeated at least three times to ensure reaction consistency. Slides were evaluated by experienced morphologists and disagreements were resolved by consensus.

RT-PCR analyses were performed to detect SARS-CoV-2. In order to specifically analyse SARS-CoV-2 RNA in the CB parenchyma, microdissections were performed on 20 µm thick paraffin-embedded sections along the margins of CB lobules. Total RNA was purified from this selected material using a RecoverAll™ Total Nucleic Acid Isolation kit (Thermo Fisher Scientific, Waltham, MA, USA) following the manufacturer’s instructions. Real-time PCR analyses were performed by using primers and TaqMan probes as previously reported (13) and run on ABI 7900HT Sequence Detection Systems (Thermo Fisher Scientific).

In CBs of negative controls, preliminary immunohistochemical analyses for nucleocapsid and Spike proteins of SARS-CoV-2 did not show any reaction (**Supplementary Figure 1**).

### Case 1

Molecular analyses did not detect SARS-CoV-2 RNA.

**Figure 1:**
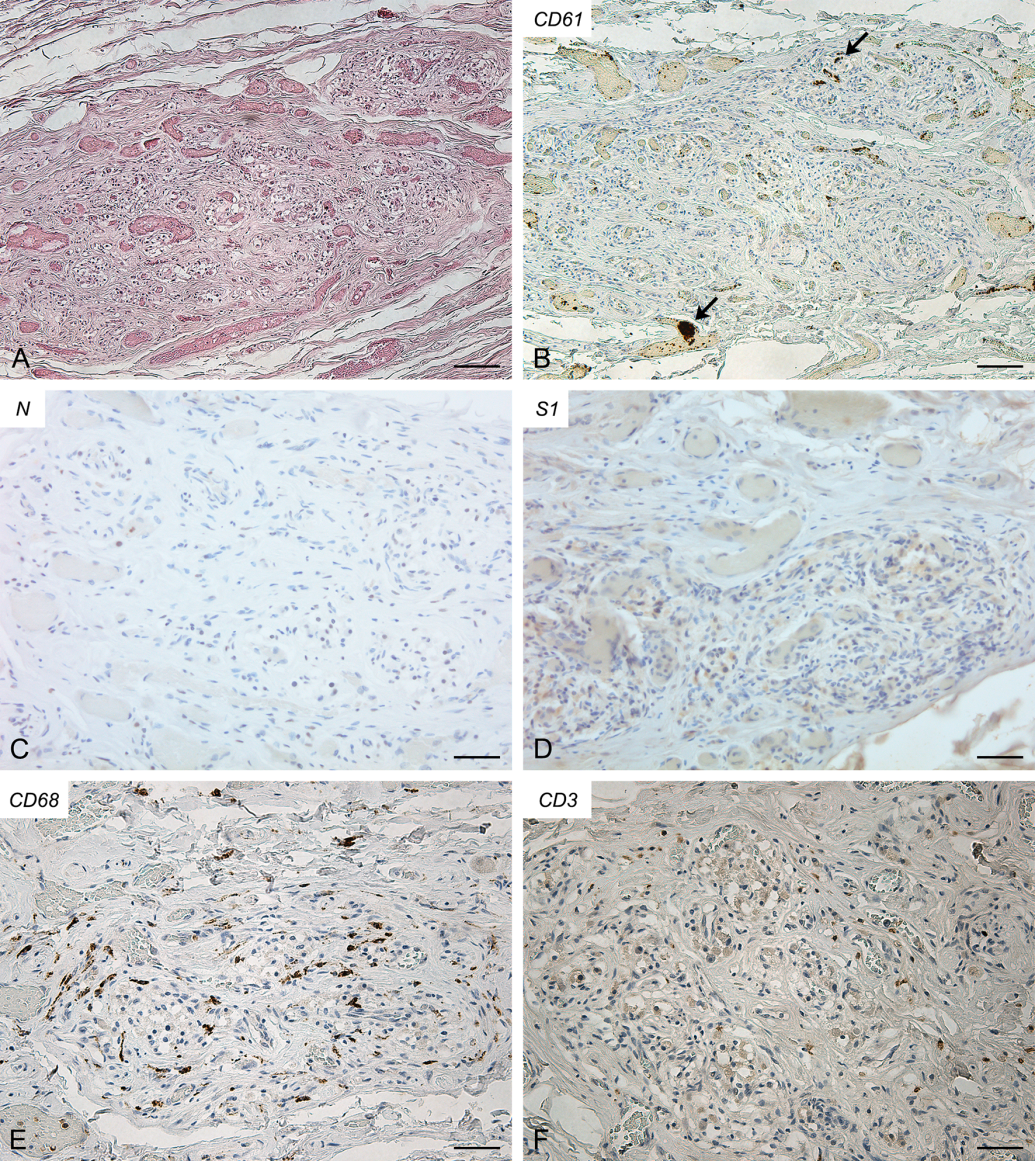
Right carotid body of Case 1 – Haematoxylin-eosin **(A)** and immunohistochemitries for CD61 (**B**; arrows: fibrin/platelet microthrombosis), nucleocapsid **(C)**, Spike **(D)**, CD68 **(E)** and CD3 **(F)**. Scale bars: 100 µm **(A, B)**; 50 µm **(C–F)**.

The right (**Figure 1**) and left (**Supplementary Figure 2**) CBs showed severe blood congestion of microvasculature, with some small CD61-positive platelet thrombi. In the left CB, focal erythrocyte extravasations were also visible in the fibroadipose tissue immediately external to the CB. Immunohistochemical stainings for SARS-CoV-2 nucleocapsid and Spike proteins were negative. Bilaterally, CD68-positive macrophages and CD3-positive lymphocytes were sparsely present in the glomic parenchyma, without aggregations. CD20-positive B lymphocytes were very rare.

### Case 2

Molecular analyses detected SARS-CoV-2 RNA in the CB (threshold cycle value: 35.42).

**Figure 2:**
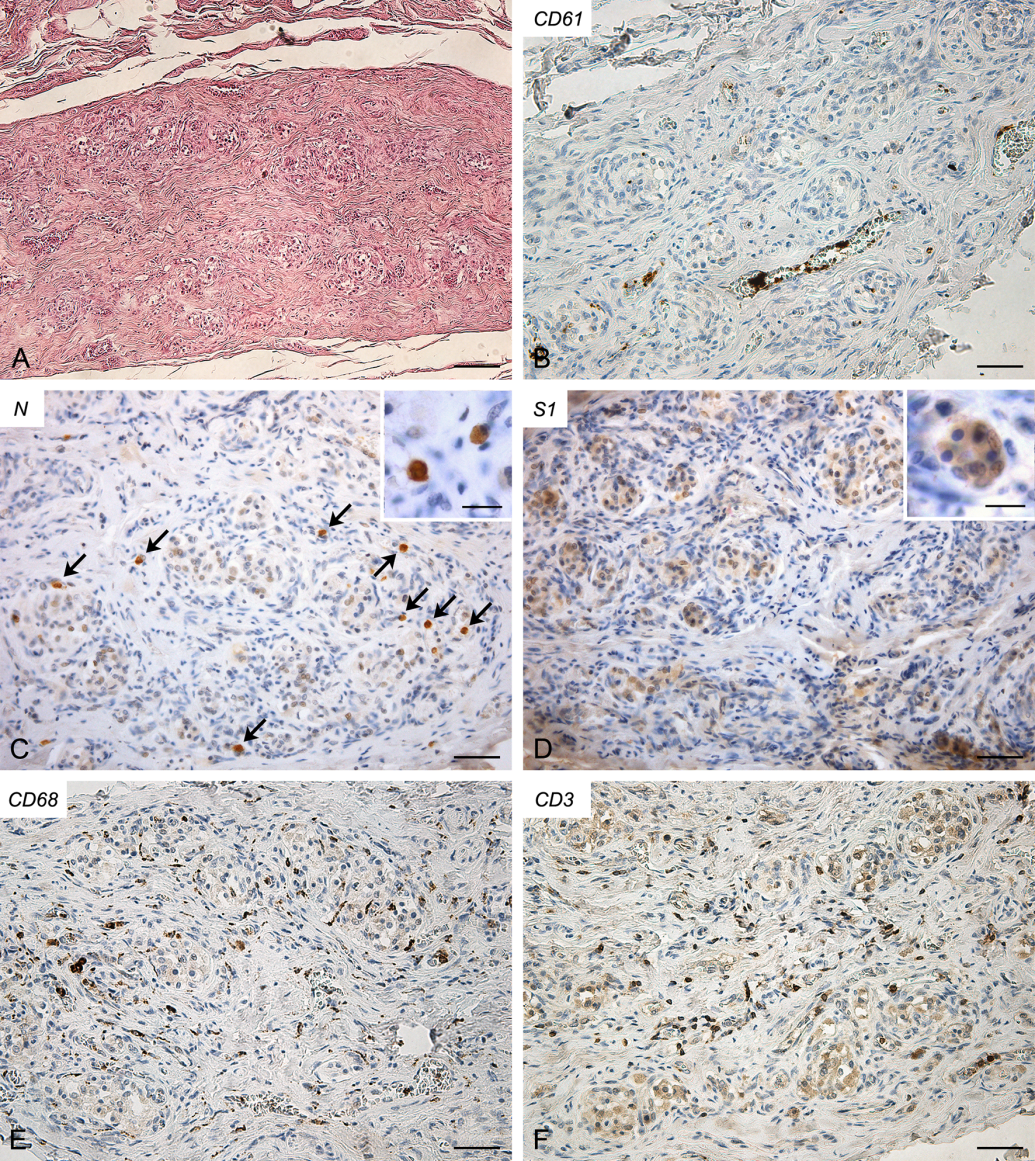
Right carotid body of Case 2 – Haematoxylin-eosin **(A)** and immunohistochemitries for CD61 **(B)**, nucleocapsid (**C**; arrows: positive cells), Spike **(D)**, CD68 **(E)** and CD3 **(F)**. Scale bars: 100 µm **(A)**; 50 µm **(B–F)**. Inserts: high magnification of positive type I cells; Scale Bar: 15 µm.

In this case (**Figure 2**), some small CD61-positive platelet thrombi were also found in the vessels of the CB, in the presence of moderate congestion. Anti-SARS-CoV-2 nucleocapsid immunohistochemistry showed some cells clearly positive in the context of glomic parenchyma. These cells were quite large and displayed roundish nuclei, consistently with type I cells. Anti-Spike immunostaining also showed positive cells in the CB, although with overall weaker immunoreactivity. CD68-positive macrophages and CD3-positive lymphocytes were diffusely present in the glomic parenchyma. CD20-positive B lymphocytes were quite rare.

### Case 3

Molecular analyses detected SARS-CoV-2 RNA in both CBs (threshold cycle value: right, 34.41; left, 35.12).

**Figure 3:**
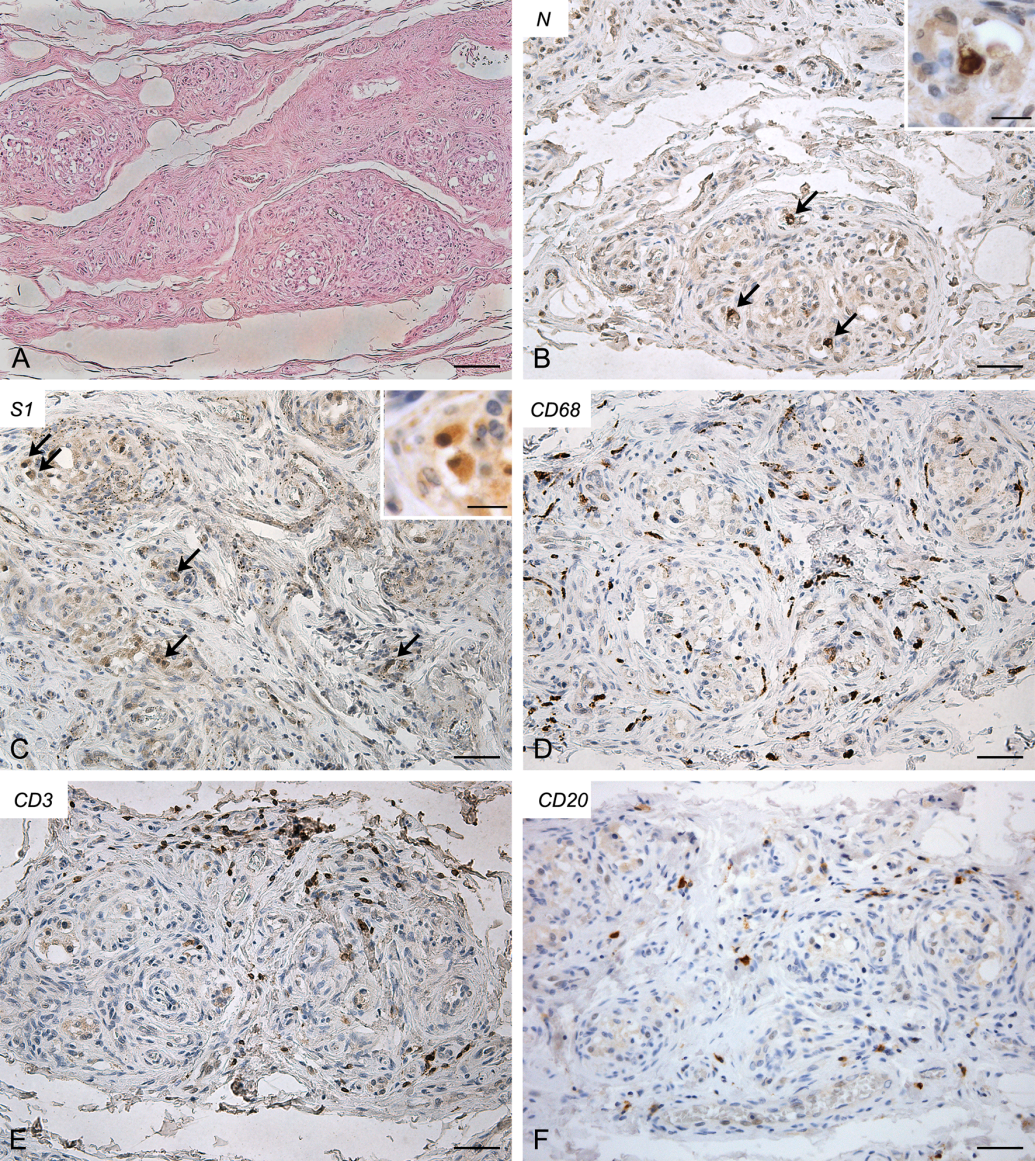
Right carotid body of Case 3 – Haematoxylin-eosin **(A)** and immunohistochemitries for nucleocapsid (**B,C**; arrows: positive cells), Spike **(D)**, CD68 **(E)** and CD3 **(F)**. Scale bars: 100 µm **(A)**; 50 µm **(B–F)**. Inserts: high magnification of positive type I cells. Scale Bar: 15 µm.

Both right (**Figure 3**) and left (**Supplementary Figure 3**) CBs showed similar features, characterized by increased connective tissue in the absence of blood congestion or microthrombosis. Anti-SARS-CoV-2 nucleocapsid immunohistochemistry evidenced some clearly positive cells in the context of glomic parenchyma. These cells were quite large and endowed with roundish nuclei, consistently with type I cells. Anti-Spike immunostaining also showed positive cells in the CB, although with weaker reaction. Immunostaining mainly involved cells with type I appearance, although positive reactions in type II cells and/or macrophages cannot be ruled out due to aspects of diffuse positivity in some slides or fields. CD68-positive macrophages were diffusely present in the glomic parenchyma. Aggregates of CD3-positive T lymphocytes were found in the CB tissue, whereas only few CD20-positive B lymphocytes were found.

### Case 4

Molecular analyses detected SARS-CoV-2 RNA in both CBs (threshold cycle value: right, 30.56; left, 33.31).

**Figure 4:**
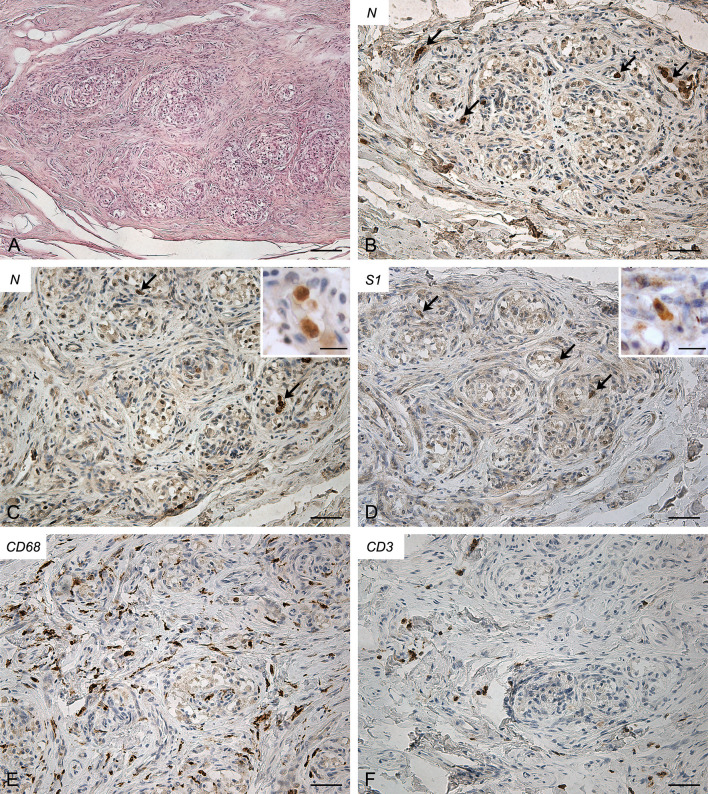
Right carotid body of Case 4 – Haematoxylin-eosin **(A)** and immunohistochemitries for nucleocapsid (**B-C**; arrows: positive cells), Spike (**D**; arrows: positive cells), CD68 **(E)** and CD3 **(F)**. Scale bars: 100 µm **(A)**; 50 µm **(B–F)**. Inserts: high magnification of positive type I cells. Scale Bar: 15 µm.

Also in this case both CBs (right, **Figure 4**; left, **Supplementary Figure 4**) exhibited similar features. They showed a slight increase in connective tissue. Blood congestion or microthrombosis were not found. Anti-SARS-CoV-2 nucleocapsid immunohistochemistry showed strongly positive cells in glomic parenchyma, bilaterally. Most of these cells were large and roundish, consistently with type I cell characteristics; however, some elongated positive cells were also found in the lobule periphery, potentially ascribable to type II cells or macrophages. Immunoreaction against SARS-CoV-2 Spike was weaker but positive cells, elongated or roundish, were visible in the glomic lobules. A high number of CD68-positive macrophages infiltrated the glomic parenchyma. Aggregates of CD3-positive T lymphocytes were also found, although less evident than in Case 2. Few CD20-positive B lymphocytes were detected.

The CBs of the five control subjects did not show microthromboses or inflammatory infiltrations (**Supplementary Figure 5**).

## Discussion

In the present study, virological and histopathological findings are shown giving evidence of the potential involvement of the CB in the complex pathological entity of COVID-19, although further analyses will be needed in larger studies.

In Case 1, RT-PCR and immunohistochemical analyses for SARS-CoV-2 nucleocapsid and Spike proteins did not allow for the identification of the virus in the CB. However, glomic vessels showed microthrombosis, blood congestion and microhaemorrhages. Apart from lungs, these findings have been reported in various organs and tissues of COVID-19 victims. Conversely, in this study, microthrombosis, blood congestion and microhaemorrhages were not found in control old subjects and, to the best of our knowledge, they have not been described before in human CB. Thus, these glomic findings may be included among the systemic effects of COVID-19, due to general mechanisms, such as the ‘cytokine storm’, coagulation dysfunction or ACE1/ACE2 imbalance, even in the absence of direct viral invasion. If confirmed, this could be particularly suggestive, as the CB is one of the structures characterized by the highest blood flow (14, 15); moreover, changes in local blood flow may play a role in chemoreception modulation.

Conversely, in Cases 2, 3 and 4, virological and immuno-histochemical analyses confirmed the direct invasion of SARS-CoV-2 in the CB. Lambermont et al. also detected SARS-CoV-2 RNA in the CB of a COVID-19 case through RT-PCR (9) but these are the first demonstrations of CB invasion through an integrated approach, including molecular (RT-PCR) and immuno-histochemical (nucleocapsid and Spike) methods. In these cases, bilateral CB involvement was found.

Concerning involved cell types, most positive cells showed clear cytological characteristics of type I cells, being roundish, large and mainly located in the center of the lobules (see inserts in **Figures 2**-**4**); however, some type II cells and macrophages were probably present among positive cells, as elongated positive-elements were also found at the lobular margins. We cannot exclude also an endothelial involvement, due to the intrinsic difficulty in identifying positive immunoreaction in the very small vessels of glomic microvasculature. Thus, while infection of type I cells is clearly evident in our immunohistochemical results, further analyses in larger casistics through double immunofluorescence will be needed to detail possible SARS-CoV-2 infection of other cell types (type II cells? endothelial cells? macrophages?) in the CB.

Immune/inflammatory cells infiltrating the CBs were also specifically evaluated. Positive CBs showed inflammatory infiltration by CD68 and CD3-positive macrophages and T lymphocytes, respectively. In particular, T lymphocytes displayed focal aggregations in the CBs with evidence of SARS-CoV-2 invasion. In our study, these findings were not found in the CB of control old subjects and they could be suggestive of specific local inflammatory reaction to SARS-CoV-2. However, we must also consider that they will need to be morphometrically confirmed in larger case series, as sparse and diffuse lympho-monocytic infiltrations have been already described in the CB, mainly in aging subjects (11, 16–20). Chronic carotid glomitis, defined by the presence of lymphocyte aggregates throughout its structure, has also been reported in subjects over the age of 50 (12, 16–20) or in opiate-related deaths (12). We cannot also theoretically exclude that comorbidities, mainly hypertension and diabetes, may have also contributed to produce or increase the inflammatory reaction. Animal experimental data are available about involvement of CB in hypertension and diabetes (21, 22), although histopathological findings in human CB have not yet been described.

In conclusion, SARS-CoV-2 direct invasion of the CB is confirmed through both immunohistochemistry and RT-PCR, with likely involvement of different cell types. We also reported histopathological findings (inflammatory aggregates, microthrombosis, blood congestion and microhaemorrhages) which could be ascribed to local and/or systemic actions of SARS-CoV-2 and which could potentially affect chemoreception.

## Data Availability Statement

The original contributions presented in the study are included in the article/**Supplementary Material**. Further inquiries can be directed to the corresponding author.

## Ethics Statement

Ethical review and approval were not required for the study on human participants in accordance with the local legislation and institutional requirements. Written informed consent for participation was not required for this study in accordance with the national legislation and the institutional requirements.

## Author Contributions

AP, RDC, VM, LB, and ES designed the study. RDC and AP performed the autopsies. AE and MC performed the histological and immunohistochemical analyses. AP, RDC, AE, and MC were responsible of histological and immunohistochemical findings interpretation. LB, SR, and AS, performed molecular analyses and interpreted the data. AP drafted the manuscript. MC prepared the figures. AP, LB, VM, and ES revised the manuscript. All authors contributed to the article and approved the submitted version.

## Funding

This work was supported by the European Union's Horizon 2020 research and innovation programme [VEO, grant number 874735].

## Conflict of Interest

The authors declare that the research was conducted in the absence of any commercial or financial relationships that could be construed as a potential conflict of interest.

## Publisher’s Note

All claims expressed in this article are solely those of the authors and do not necessarily represent those of their affiliated organizations, or those of the publisher, the editors and the reviewers. Any product that may be evaluated in this article, or claim that may be made by its manufacturer, is not guaranteed or endorsed by the publisher.

## Supplementary Material

Supplementary Figure 1 Negative controls for anti-nucleocapsid **(A)** and -Spike **(B)** immunohistochemistries in human carotid bodies from autopsy cases predating COVID-19 pandemic. Positive controls for anti-nucleocapsid **(C)** and -Spike **(D)** immunohistochemistries in human lung samples from ascertained COVID-19 victims. Scale bars: 50 µm **(A, B)**; 100 µm **(C, D)**.

Supplementary Figure 2 Left carotid body of Case 1 – Haematoxylin-eosin **(A)** and immunohistochemitries for CD61 (**B**; arrow: fibrin/platelet microthrombosis), nucleocapsid **(C)**, Spike **(D)**, CD68 **(E)** and CD3 **(F)**. Scale bars: 100 µm **(A, B)**; 50 µm **(C–F)**.

Supplementary Figure 3 Left carotid body of Case 3 – Haematoxylin-eosin **(A)** and immunohistochemitries for nucleocapsid **(B)**, Spike **(C)**, CD68 **(D)**, CD3 **(E)** and CD20 **(F)**. Scale bars: 100 µm **(A, F)**; 50 µm **(B–E)**.

Supplementary Figure 4 Left carotid body of Case 4 – Haematoxylin-eosin **(A)** and immunohistochemitries for nucleocapsid (**B–C**; arrows: positive cells), Spike (**D**; arrows: positive cells), CD68 **(E)** and CD3 **(F)**. Scale bars: 100 µm **(A)**; 50 µm **(B–F)**.

Supplementary Figure 5 Carotid body of control old subjects – Immunohistochemitries for CD61 **(A)**, CD68 **(B)**, CD3 **(C)** and CD20 **(D)**. Scale bars: 50 µm **(B–D)**.
