# Supplementary figures and images for: Case Report: The Carotid Body in COVID-19: Histopathological and Virological Analyses of an Autopsy Case Series
Source: Front Immunol. 2021 Oct 26;12:736529. doi: 10.3389/fimmu.2021.736529 (PMC8576382; doi:10.3389/fimmu.2021.736529)

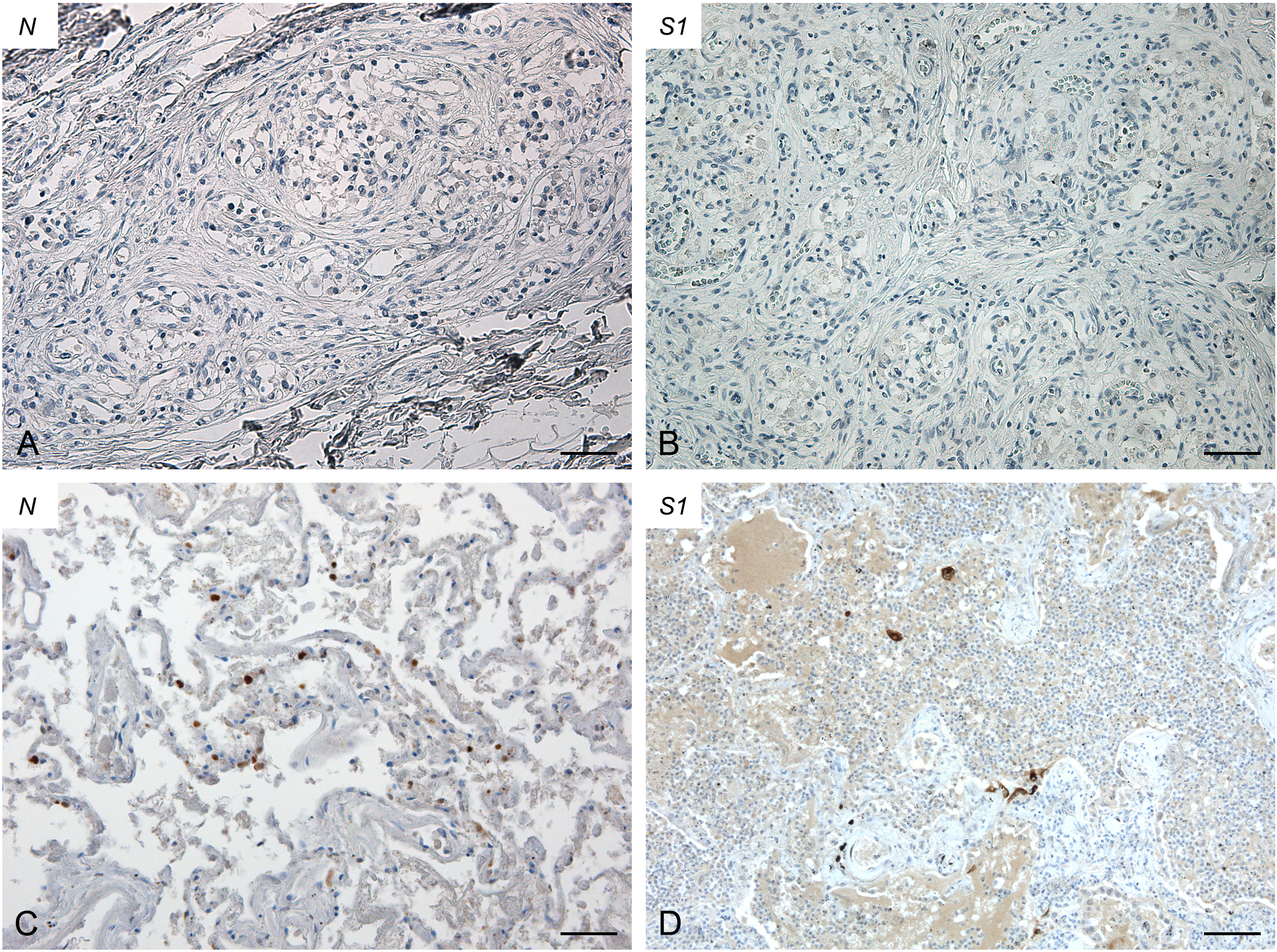

Supplement: Supplementary Figure 1 — Negative controls for anti-nucleocapsid (A) and -Spike (B) immunohistochemistries in human carotid bodies from autopsy cases predating COVID-19 pandemic. Positive controls for anti-nucleocapsid (C) and -Spike (D) immunohistochemistries in human lung samples from ascertained COVID-19 victims. Scale bars: 50 µm (A, B); 100 µm (C, D). [file Image_1.tif]

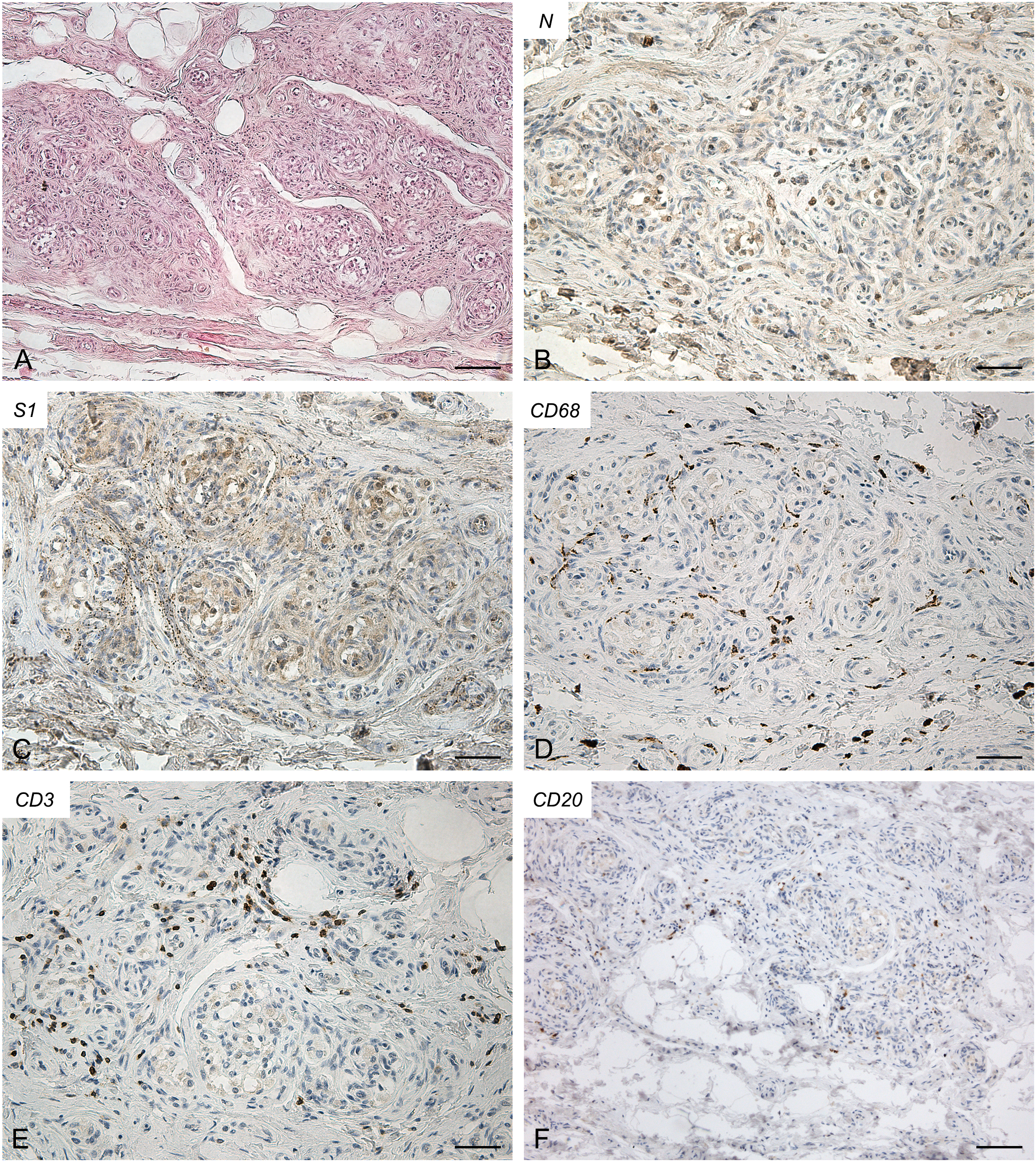

Supplement: Supplementary Figure 3 — Left carotid body of Case 3 – Haematoxylin-eosin (A) and immunohistochemitries for nucleocapsid (B), Spike (C), CD68 (D), CD3 (E) and CD20 (F). Scale bars: 100 µm (A, F); 50 µm (B–E). [file Image_3.tif]

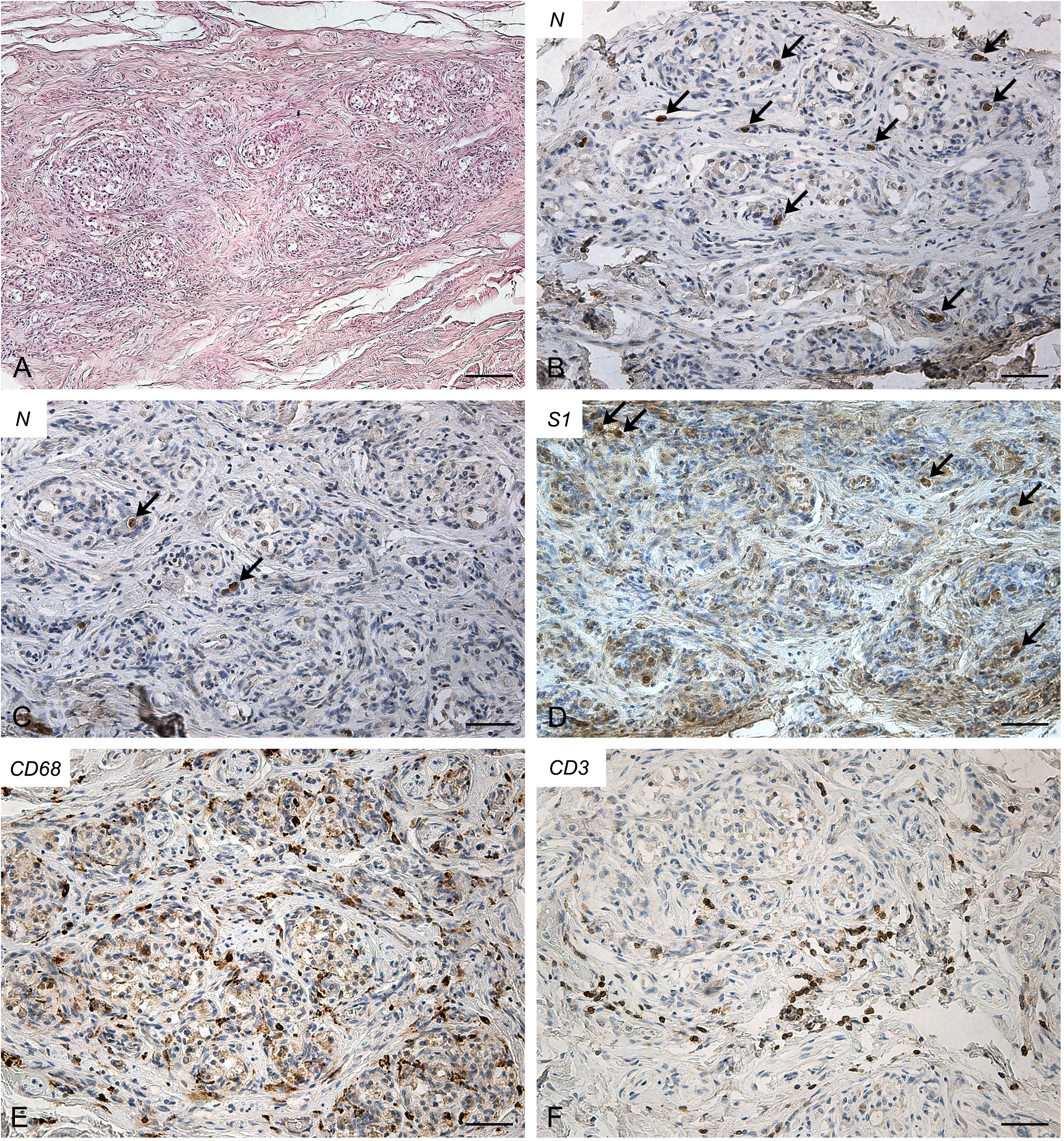

Supplement: Supplementary Figure 4 — Left carotid body of Case 4 – Haematoxylin-eosin (A) and immunohistochemitries for nucleocapsid (B–C; arrows: positive cells), Spike (D; arrows: positive cells), CD68 (E) and CD3 (F). Scale bars: 100 µm (A); 50 µm (B–F). [file Image_4.tif]

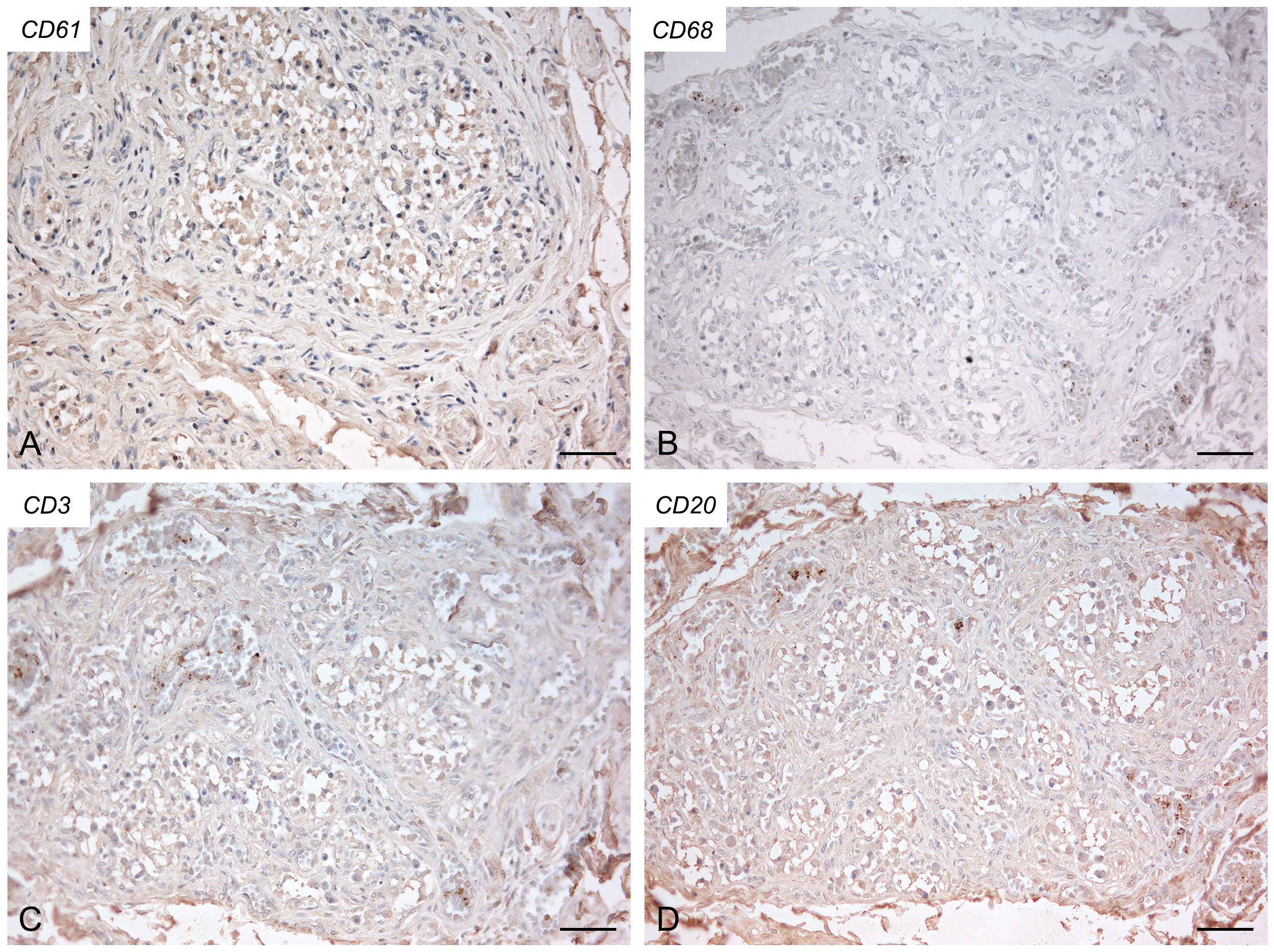

Supplement: Supplementary Figure 5 — Carotid body of control old subjects – Immunohistochemitries for CD61 (A), CD68 (B), CD3 (C) and CD20 (D). Scale bars: 50 µm (B–D). [file Image_5.tif]
