# Supplementary material for: Case Report: The Carotid Body in COVID-19: Histopathological and Virological Analyses of an Autopsy Case Series
Source: Front Immunol. 2021 Oct 26;12:736529. doi: 10.3389/fimmu.2021.736529 (PMC8576382; doi:10.3389/fimmu.2021.736529)
Supplement: Supplementary file 6 [file Table_1.docx]

**Supplemental 1.** Timeline table. Clinical and pathological characteristics of autopsy cases.

| Case | Age | Gender | Interval between positive test and death (days) | Comorbidities | Clinical course | Autopsy and histopathological findings |
| --- | --- | --- | --- | --- | --- | --- |
| 1 | 72 | Male | 1 | - | Recovery for dyspnoea  Initially negative for SARS-CoV-2  Death for respiratory failure after SARS-CoV-2 positivization | Lung:  - Diffuse alveolar damage  - Lung vascular congestion  - Platelet/fibrin microthrombosis  - Alveolar and subpleural haemorrhagic infiltrations  - Lymphomonocytic infiltrations  Other organs:  - Spleen white pulp depletion  - Renal congestion and lymphomonocytic infiltrations  - Congestion, platelet/fibrin microthrombosis and microhaemorrhages in brain |
| 2 | 89 | Female | 16 | Hypertension  Diabetes  Vascular dementia | Death for respiratory failure  Non-invasive O_2_ supply only few hours before death | Lung:  - Chronic emphysema  - Diffuse alveolar damage  - Lung vascular congestion  - Platelet/fibrin microthrombosis  - Alveolar and subpleural haemorrhagic infiltrations  - Lymphomonocytic infiltrations  Other organs:  - Pericardial lymphomonocytic infiltrations  - Spleen subcapsular haemorrhages  - Renal congestion and lymphomonocytic infiltrations |
| 3 | 77 | Female | 2 | Hypertension  Chronic renal insufficiency | Recovery for dyspnoea and asthenia due to heart failure  *Klebsiella pneumoniae* pneumonia  Hospital-acquired SARS-CoV-2 infection  Death for respiratory failure | Lung:  - Chronic emphysema  - Diffuse granulocytic infiltration (bacterial pneumonia)  - Diffuse alveolar damage  - Lung vascular congestion  - Platelet/fibrin microthrombosis  - Alveolar haemorrhagic infiltrations  - Focal lymphomonocytic infiltrations  Other organs:  - Spleen subcapsular haemorrhages and white pulp depletion  - Renal congestion, lymphomonocytic infiltrations and corpuscles degeneration  - Adrenal lymphomonocytic infiltrations |
| 4 | 84 | Male | 20 | Hypertension  Parkinson’s disease  Vascular dementia | Recovery and death for respiratory failure | Lung:  - Chronic emphysema  - Diffuse alveolar damage  - Lung vascular congestion  - Platelet/fibrin microthrombosis  - Alveolar and subpleural haemorrhagic infiltrations  - Lymphomonocytic infiltrations |
